# Preservation of cfRNA in cytological supernatants for cfDNA & cfRNA double detection in non‐small cell lung cancer patients

**DOI:** 10.1002/cam4.70197

**Published:** 2024-09-05

**Authors:** Yidan Ma, Yifei Wang, Lei He, Jun Du, Lin Li, Zhixin Bie, Yuanming Li, Xiaomao Xu, Wei Zhou, Xiaonan Wu, Li Yang, Jing Di, Chenyang Li, Xiaoguang Li, Dongge Liu, Zheng Wang

**Affiliations:** ^1^ Department of Pathology, Beijing Hospital, National Center of Gerontology Institute of Geriatric Medicine, Chinese Academy of Medical Sciences Beijing People's Republic of China; ^2^ Department of Oncology, Beijing Hospital, National Center of Gerontology Institute of Geriatric Medicine, Chinese Academy of Medical Sciences Beijing People's Republic of China; ^3^ Department of Minimally Invasive Tumor Therapies Center, Beijing Hospital, National Center of Gerontology Institute of Geriatric Medicine, Chinese Academy of Medical Sciences Beijing People's Republic of China; ^4^ Department of Respiratory and Critical Care Medicine, Beijing Hospital, National Center of Gerontology Institute of Geriatric Medicine, Chinese Academy of Medical Sciences Beijing People's Republic of China

**Keywords:** cell‐free RNA, driver gene, fusion, liquid biopsy, NSCLC

## Abstract

**Backgroud:**

Supernatants from various cytological samples, including body cavity effusion, sputum, bronchoalveolar lavage fluid (BALF), and needle aspiration, have been validated for detecting genetic alterations using cell‐free DNA (cfDNA) in patients with non‐small cell lung cancer (NSCLC). However, the sensitivity of fusion variations detection remains challenging. The protection of cell‐free RNA (cfRNA) is critical for resolving the issue.

**Methods:**

A protective solution (PS) was applied for preserving cfRNA in cytological supernatant (CS), and the quality of protected cfRNA was assessed by cycle threshold (CT) values from reverse transcription quantitative polymerase chain reaction (RT‐qPCR). Furthermore, we collected an additional set of malignant cytological and matched tumor samples from 84 NSCLC patients, cfDNA & cfRNA extraction and double detection for driver gene mutations was validated using the multi‐gene mutations detection by RT‐qPCR.

**Results:**

Under the optimal protection system, 91.0% (101/111) of cfRNA were protected effectively. Among the 84 NSCLC patient samples, seven cytological samples failed the tests. In comparison with tumor samples, the overall sensitivity and specificity of detecting driver genes of supernatant cfDNA and cfRNA were 93.8% (74/77) and 100% (77/77), respectively. Notably, when focusing exclusively on patients with fusion gene changes, both sensitivity and specificity reached 100% (11/11) for EML4‐ALK, ROS1, RET fusions, and MET ex14 skipping.

**Conclusion:**

These findings suggest that cfDNA & cfRNA extraction and double detection strategy recommended in this study improve the accuracy of driver genes mutations test, especially for RNA‐based assay.

## Graphical abstract

A protective solution was applied for preserving cfRNA in cytological supernatant. These findings suggest that cfDNA and cfRNA extraction and double detection strategy recommended in this study improve the accuracy of driver genes mutations test, especially for RNA‐based assay.

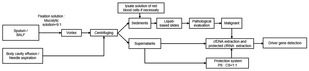

## 1 INTRODUCTION

Primary lung cancer is one of the most prevalent malignancies worldwide and a leading cause of cancer‐related mortality, which is primarily classified into two major histological subtypes: small cell lung cancer (SCLC) and non‐small cell lung cancer (NSCLC).^1^, ^2^, ^3^ Current clinical guidelines for NSCLC recommend a comprehensive molecular profiling encompasses 11 driver genes, including EGFR mutations, ALK, KRAS, ROS1, BRAF, NTRK1/2/3, MET ex14 skipping, RET, and ERBB2 (HER2). In addition to traditional tumor specimens, recent studies have explored the use of various cytological supernatant (CS) samples for molecular analysis, such as body cavity effusion, sputum, bronchoalveolar lavage fluid (BALF), and needle aspiration.^4^, ^5^, ^6^, ^7^, ^8^

While liquid biopsy samples for cell‐free DNA (cfDNA) testing offer several obvious advantages, such as non or minimally invasion, ease of obtaining, and high sensitivity for single nucleotide variations (like point mutations, deletions, and insertions), they are short in detecting structural variations such as gene fusions, which significantly affects their detection sensitivity.^9^, ^10^, ^11^

Fusion alternations of ALK, ROS1, RET and NTRK1/2/3 are recommended for testing in NSCLC.^3^, ^12^ Previous researches indicates that RNA level sequencing outperforms DNA sequencing in identifying fusion genes.^13^, ^14^ DNA & RNA double test has been explored in tumor samples by next generation sequencing (NGS) and reverse transcription quantitative polymerase chain reaction (RT‐qPCR), etc.^15^ Cell‐free RNA (cfRNA) has not been verified fully for gene fusion detection and may face challenges for analysis in CS samples.

This study introduces a novel approach aimed at protecting cfRNA in various CS samples. We have developed a protocol for cfDNA and cfRNA extraction and double detection in CS samples to improve the accuracy of driver genes mutations by RT‐qPCR test in NSCLC.

## 2 MATERIALS AND METHODS

### 2.1 Study design

This study was conducted with samples collected from Beijing Hospital between November 2022 and September 2023. A total of 111 CS samples were enrolled in the study, including 26 body cavity effusions, 16 sputum samples, 60 bronchoalveolar lavage fluids (BALF), and 9 needle aspirations. These samples were utilized to assess the efficacy of a laboratory developed cfRNA protective solution (PS) (patent application number 202310823963.0, China National Intellectual Property Administration) using reverse transcription quantitative polymerase chain reaction (RT‐qPCR). The study aimed to establish the optimal proportion for mixing CS with cfRNA PS and to evaluate the quality of the protected cfRNA.

**FIGURE 1:**
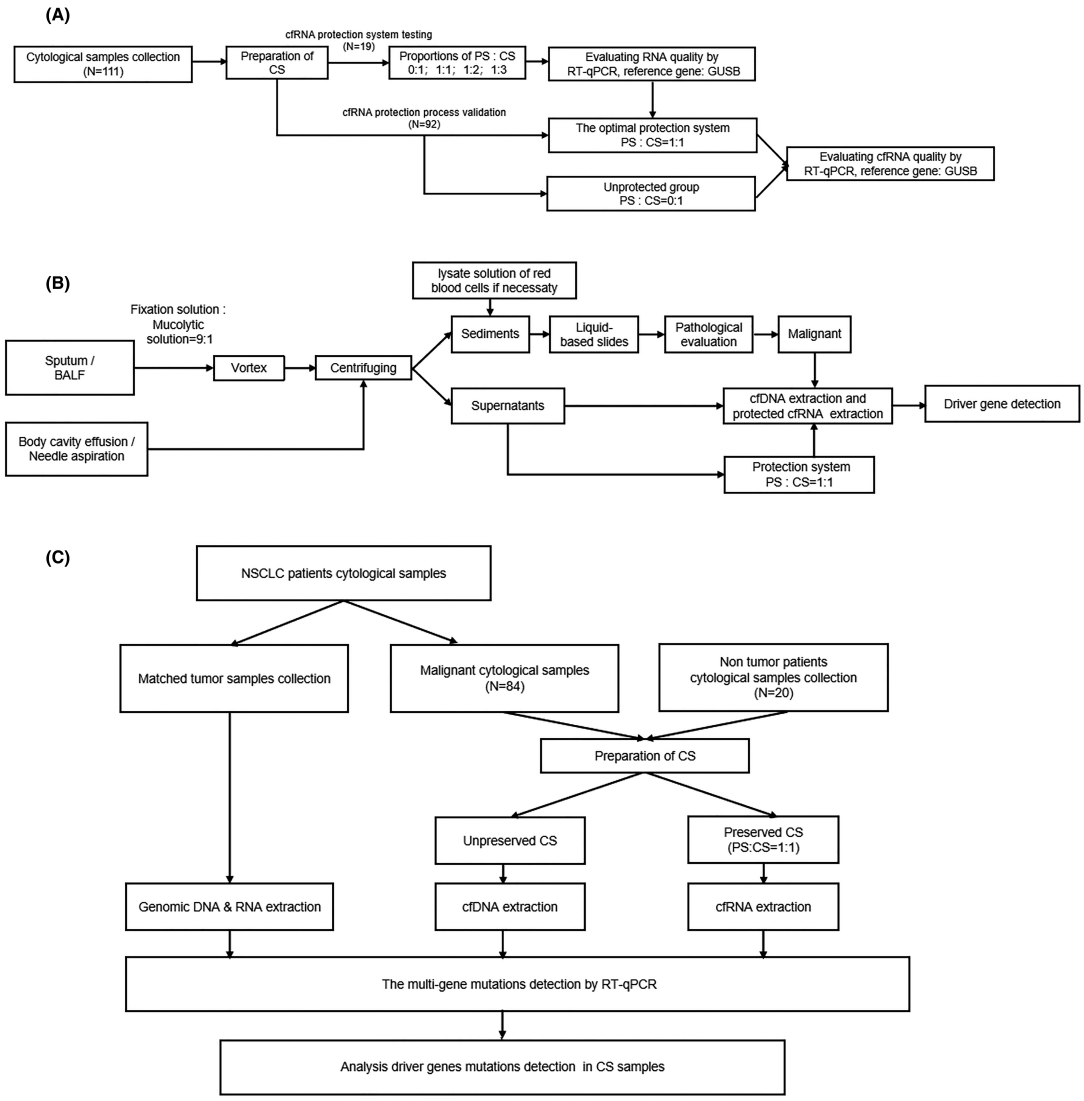
Flowcharts of study design. (A) cfRNA protection system of testing and validation set; (B) processing of preparation for CS samples; (C) detection of 11 driver genes mutations in CS and matched tumor samples. CS, cytological supernatant; PS, protective solution.

Additionally, malignant CS samples and matched tumor samples were collected from 84 NSCLC patients, along with 20 non‐cancer cytological samples and their mached samples (including pneumonia, chronic obstructive pulmonary diseases, bronchiectasis, asthma, interstitial pneumonia, pulmonary abscess, sarcoidosis, hemoptysis, and heart failure) for genotyping purposes. The inclusion criteria for non‐cancer cohort were any individuals of age ≥18 who presented in Beijing Hospital. Patients diagnosed with any type of malignant tumor before or at the time of enrollment were excluded. The study was aimed at validating the feasibility and accuracy of preserved cfRNA in genetic analysis. The details of the study design, including the process and selection criteria, are illustrated in Figure 1A,C.

Patients with fusions in cfRNA level had a clinical follow‐up until disease progression or May 2024. Tumor response to treatment was assessed according to the Response Evaluation Criteria in Solid Tumors (version 1.1). Progression‐free survival (PFS) was defined as the time from initiation of targeted therapy to date of documented disease progression. Objective response rate (ORR) was defined as the proportion of patients with a complete response (CR) or partial response (PR) to treatment according to RECIST. Disease control rate (DCR) was defined as the percentage of CR, PR, and stable disease (SD).

Ethical approval for this study was approved by the Beijing Hospital Human Committee (approval number 2020BJYYEC‐065–02). All participants provided written informed consent.

### 2.2 Preparation of supernatants from cytological samples

In this study, cytological samples were categorized into two groups based on their mucus content (Figure 1B). For samples containing mucus, such as sputum and BALF, an initial mucolysis treatment was applied. This involved adding 3–5 mL of sputum or BALF to a fixation liquid composed of 2% polyethylene glycol 400 (Aladdin) in 50% Ethyl Alcohol (fixation solution), combined with a mucolytic solution of 0.5 mol/L dithiothreitol (Sigma) in a 9:1 ratio (laboratory developed, patent application number 202310823963.0, China National Intellectual Property Administration), followed by thorough vortex. For samples without mucus, 20 mL needle aspiration samples suspended in the above fixation solution and 20–50 mL body cavity effusions were collected in each centrifuge tube. Subsequently, all cytological samples underwent centrifugation. The sediments were re‐suspended in PreservCyt Solution (Hologic Inc.) to prepare liquid‐based slides for pathological evaluation. When malignant cells were found in cytological samples, the CS samples were collected for further genotyping. Detailed preanalytical steps were described in the supplementary materials.

### 2.3 Protection of cf RNA in CS samples

A subset of 19 CS samples was further divided into four volumes: 6, 4.5, 4, and 3 mL. To these, 0, 1.5, 2, and 3 mL of cfRNA PS containing 4 mol/L guanidine isothiocyanate were added, respectively. This resulted in PS:CS proportions of 0:1, 1:1, 1:2, and 1:3 for the four groups. Based on the cycle threshold (CT) values and amplification curves obtained from RT‐qPCR analysis of the preserved cfRNA in these groups, the optimal PS:CS proportion was determined.

A total of 92 CS samples were collected for a further validation test, in comparison of the CT values and the amplification curves between the proportion of PS:CS = 0:1 and 1:1 group.

### 2.4 Extraction of cfDNA, cfRNA, and genomic DNA & RNA

In this phase of the study, cfDNA and cfRNA were extracted from 4 mL samples of CS using the Circulating DNA Extraction Kit (Amoy Diagnostics). Post‐extraction, the cfDNA and cfRNA were eluted in 40 μL of the elution buffer and subsequently stored at −80°C pending genotyping analysis.

Parallel to this, genomic DNA and RNA were extracted from tumor samples employing the FFPE DNA Extraction Kit and the FFPE RNA Extraction Kit (Amoy Diagnostics), following the manufacturer's instructions. The concentrations of the purified cfDNA and genomic DNA were estimated by the QuantiFluor dsDNA (Promega).

### 2.5 Evaluation of quality of extracted cfRNA

The extracted cfRNA was performed with reverse transcription (RT) using the RT reagent kit (Amoy Diagnostics). The synthesized complementary DNA was undergone RT‐qPCR analysis (Amoy Diagnostics). Glucuronidase Beta (GUSB, a house‐keeping gene) was selected as the reference gene to assess the quality of cfRNA, with the evaluation based on RT‐qPCR's amplification curves and CT values.^16^, ^17^ Amplification was performed with Stratagene Mx3000PTM (Agilent). An evaluation system was set that the CT values ≤25.0 was defined as successful cfRNA protection, CT values between 25.0 and 27.0 were gray zone, CT values >27.0 or no amplification curves was defined as failed cfRNA protection.

### 2.6 Detection of 11 driver gene mutations in CS samples and matched samples

Respectively, cfDNA and cfRNA obtained from CS samples in 84 NSCLC cases and 20 non tumor samples, 11 driver genes mutations were detected by using Pan Lung Cancer PCR Panel kit (Amoy Diagnostics),^18^ according to the manufacturer's instructions (Figure 1C). EGFR mutation, KRAS, BRAF, and ERBB2 (HER2) were detected at the cfDNA level, while ALK, ROS1, NTRK1/2/3, RET, and MET ex14 skipping mutations were detected at the cfRNA level.

A total of 11 driver genes mutations were detected in matched tumor and non‐tumor samples using the Pan Lung Cancer PCR Panel kit (Amoy Diagnostics), according to the manufacturer's instructions (Figure 1C). The criteria used to establish the positivity of samples was followed the manufacturer's instruction.

### 2.7 Statistical analysis

All statistical analyses were conducted using the R statistical programming language. The paired Wilcoxon signed‐rank test was applied to evaluate differences in samples treated with four different proportions of PS:CS. *p*‐values were calculated to compare data from NSCLC and non‐cancer group for the following variables: mean ages using student's unpaired two‐tailed *t*‐tests, and sex distribution using a chi‐squared test. *p* < 0.05 was considered statistically significant (two‐sided). Kaplan–Meier estimation was used to designate PFS.

Graphic analyses were performed using GraphPad Prism 8.0 software (GraphPad Software).

## 3 RESULTS

### 3.1 Evaluation of cfRNA protective solution efficiency of preserved cfRNA

**FIGURE 2:**
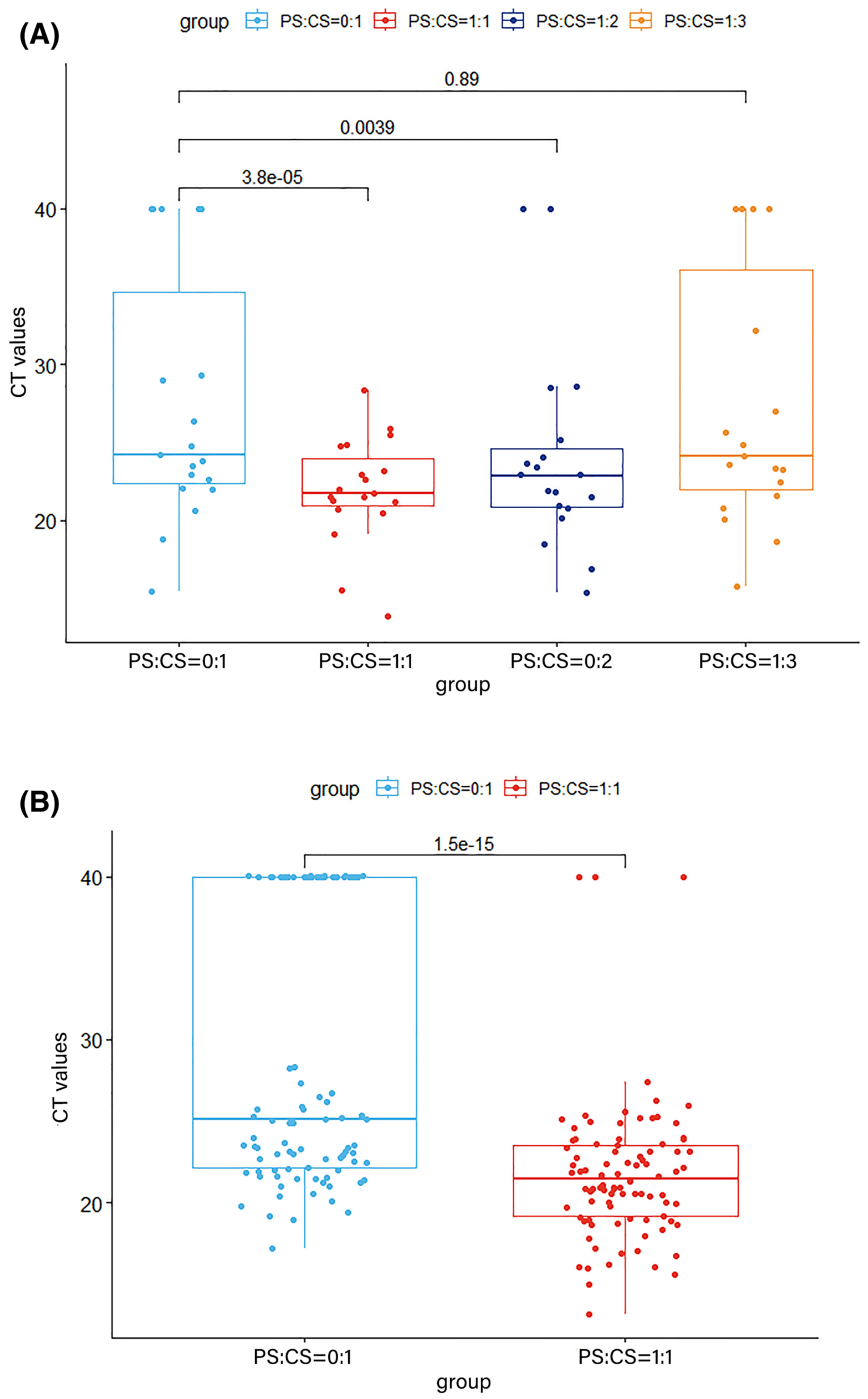
Exploration and validation the optimal proportions of PS:CS for cfRNA protection. CS samples were protected with different proportions of PS:CS before cfRNA extraction. The efficacy of cfRNA PS was evaluated through RT‐qPCR CT values across different PS:CS proportions. Paired Wilcoxon signed‐rank test was used for evaluation. (A) CT values for cfRNA in PS:CS proportions of 0:1, 1:1, 1:2, and 1:3 in 19 CS samples; (B) 92 CS samples for validation with the proportion of PS:CS = 1:1 and 0:1.

The efficacy of cfRNA PS was evaluated through RT‐qPCR CT values across different PS:CS proportions in 19 CS samples. The median CT values for cfRNA in PS:CS proportions of 0:1, 1:1, 1:2, and 1:3 were 24.18, 21.68, 22.87, and 23.14, respectively. The CT values in the 1:1 and 1:2 groups were significantly better than the 0:1 (*p* < 0.001; *p* = 0.004), while no significant difference was observed between the 1:3 and 0:1 group (*p* > 0.05) (Figure 2A). Additionally, 89.5% (17/19, 1:1 group), 73.7% (14/19, 1:2 group), 47.4% (9/19, 1:3 group) samples were superior to their paired unprotected CS samples (0:1 group) in comparison with CT values. 68.4% (13/19, 1:1 group), 57.9% (11/19, 1:2 group), 31.6% (6/19, 1:3 group) achieved the CT value equal or less than 23.0. The variability of CT values (≤25.0, ≤27.0, and >27.0 or no amplification curves) for determining protected ratios is presented in Table 1A. Based on these results, the optimal PS:CS proportion was 1:1.

**TABLE 1:** Protected ratios of cfRNA in the groups with various proportions of PS:CS under the different CT values (A) Testing set (*N* = 19); (B) validation set (*N* = 92).

CT value				
Proportion of PS:CS	≤23.0	≤25.0	≤27.0	>27.0 or no amplification curve detected
(A)				
0:1	7/19 (36.8%)	11/19 (57.9%)	12/19 (63.2%)	7/19 (36.8%)
1:1	13/19 (68.4%)	16/19 (84.2%)	18/19 (94.7%)	1/19 (5.3%)
1:2	11/19 (57.9%)	14/19 (73.7%)	15/19 (78.9%)	4/19 (21.1%)
1:3	6/19 (31.6%)	11/19 (57.9%)	13/19 (68.4%)	6/19 (31.6%)
(B)				
0:1	33/92 (35.9%)	46/92 (50.0%)	57/92 (62.0%)	35/92 (38.0%)
1:1	63/92 (68.5%)	81/92 (88.0%)	88/92 (95.7%)	4/92 (4.3%)

92 CS samples were included in validation set for comparing the successfully protected ratios of 0:1 and 1:1 group. The median CT value of cfRNA was 21.45 in the 1:1 group, significantly superior to that of 0:1 (25.08; *p <* 0.001) (Figure 2B). 91.3% (84/92) CT values of cfRNA in 1:1 group were superior to cfRNA in 0:1 group, and it was 91.0% (101/111) in all 111 samples. 35.9% (33/92, 0:1 group) and 68.5% (63/92, 1:1 group) samples met the CT value equal or less than 23.0 in validation set. Analysis data of different CT values in validation set was displayed in Table 1B. Amplification curves of protected cfRNA in the groups with various proportions of PS:CS by RT‐qPCR were shown in Figure S1.

### 3.2 Patient characteristics in NSCLC group

The clinicopathologic characteristics of the 84 NSCLC patients enrolled in the study are summarized in Table 2. The median age was 70.5 years (range 39–92), with a majority of patients being male (60.7%). Complete TNM staging information was available for 73 (86.9%) patients, most of whom were in late‐stage (IIIB to IV) disease (91.8%). Treatment details were available for 58 patients (69.0%), with 42 (50.0%) being treatment‐naive and 16 (19.1%) having relapsed from prior treatment. For the 20 enrolled non‐cancer patients, the mean age was 62.4 years (range 31–85), which was younger than NSCLC patients (*p* = 0.017). 60.0% non‐cancer patients were male, with no statistically significant to the NSCLC patients (*p* = 0.953).

**TABLE 2:** Clinicopathologic characteristics of 84 enrolled NSCLC patients.

		Enrolled patients (*N* = 84)	(%) (total)
Age‐median, (range)	Median	70.5 [39–92]	
Sex‐no (%)	Male	51	(60.7)
	Female	33	(39.3)
Stage‐n (%)	I–IIIA	6	(7.1)
	IIIB–IV	67	(79.8)
	Unknown	11	(13.1)
Sample	Body cavity effusion	36	(42.8)
	Sputum	20	(23.8)
	BALF	20	(23.8)
	Needle aspiration	8	(9.5)
Patients' treatment status	Treatment naive	42	(50.0)
	Relapsed from prior treatment	16	(19.0)
	Unknown	26	(31.0)

### 3.3 Analysis of driver gene detection performance in cytological samples

Eleven driver genes mutations were detected in 84 malignant CS samples with NSCLC. All cfRNA used for genotyping were protected using a PS:CS proportion of 1:1. In total, 91.7% (77/84) CS samples were tested successfully, including 100% (20/20; 36/36) in sputum and body cavity effusion CS samples, 85% (17/20) in BALF and 50% (4/8) in needle aspiration suspension samples respectively. In 7 failed samples, CT values were greater than 27 or no amplification curves. The included sample types for the present study are the main cytological sources in our medical center, except for these types, the available sample sizes of cerebrospinal fluid and fine needle aspiration from NSCLC patients are relative limited. The clinicopathologic characteristics of seven samples with failed detection are shown in Table S1.

None of the driver genes were positive in the 20 non‐cancer patients, confirming 100% specificity in testing cfDNA and cfRNA.

**FIGURE 3:**
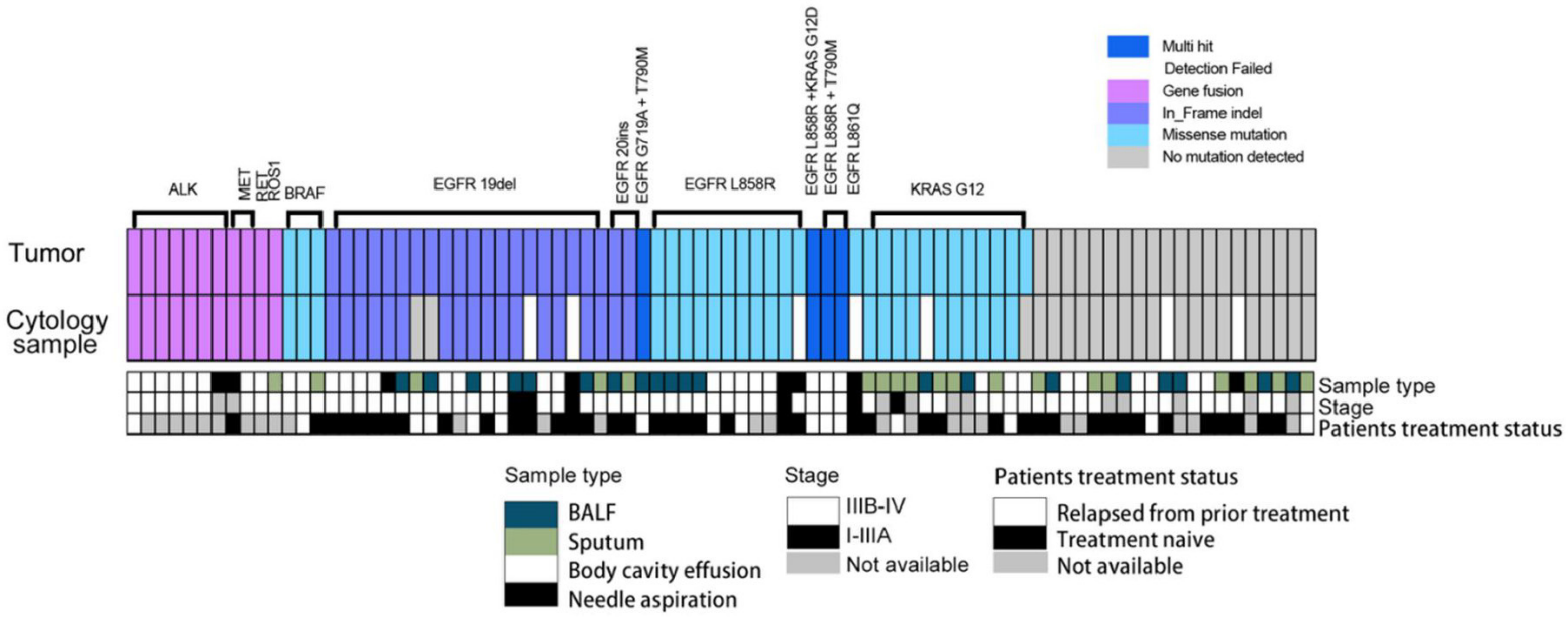
Hotspot mutations detection profile in cytological supernatant compared to tumor samples. Hotspot mutations detection profile in cytological supernatant compared to tumor samples. (Top) Plot shows the mutation profiles, the colors indicate the mutation types. (Bottom) Plot illustrates the sample type, patients stage and treatment status. Multi_Hit indicates multi types of variation.

Among the 77 successfully tested NSCLC CS samples, overall sensitivity and specificity were 93.8% (74/77) and 100% (77/77). At DNA level, driver genes positive rate was 48 (62.3%) in the tumor samples, and 45 (58.4%) in CS samples for SNV/InDels detection, resulting in a total sensitivity of 93.8% (45/48) and specificity of 100% (48/48). The most common mutation types detected at the cfDNA level was EGFR exon 19 deletion (20.8%, *n* = 16), followed by EGFR exon 21 L858R mutation (16.9%, *n* = 13). Regarding RNA level, 11 (14.3%) samples were detected positive in matched tumor samples, with the same results in CS samples for fusion testing, demonstrating 100% sensitivity and specificity. EML4‐ALK fusion was identified in seven patients (9.1%, 7/77), along with 2 MET ex14 skipping mutations, 1 ROS1 gene fusion, and 1 RET gene fusion (Figure 3). The clinicopathologic characteristics of 11 samples with genes alternations in cfRNA level are shown in Table S2. One case with ROS1 gene fusion positive from sputum CS sample is displayed Figure S2.

The sensitivity of CS samples varied with 94.1% for BALF, 97.2% for body cavity effusion, 100% for needle aspiration, and 95.0% for sputum. For different stages, the sensitivity of I–IIIA stages was 100%, while IIIB–IV was 95.2%. In terms of treatment history, sensitivity was 97.1% for treatment‐naive, 87.5% for relapsed from prior treatment cases.

### 3.4 Follow‐up of patients with gene fusions

The clinicopathologic characteristics and follow‐up information of 11 samples with fusions in cfRNA level are shown in Table S2. The median follow‐up was 8 months (4–14 months). The median PFS was 14 months (*n* = 10). Among all the 10 patients with targeted therapy, the ORR was 60% and the DCR was 100%.

## 4 DISCUSSION

Previous studies have proved the efficacy of liquid biopsy samples to be effective surrogate specimens for the driver genes mutations detection in NSCLC, particularly when tumor samples are difficult to obtain or insufficient for comprehensive molecular testing.^19^, ^20^, ^21^, ^22^, ^23^, ^24^, ^25^ While cfDNA‐based assays have validated sensitive enough in detecting SNV/InDels, their effectiveness in identifying gene fusion alterations remains problematic.^26^, ^27^ Furthermore, cfRNA‐based detection faces challenges mainly related to the quality of cfRNA.^28^

We describe a protocol which can protect the quality of cfRNA from various types of CS samples, including of body cavity effusions, sputa, BALF and needle aspirations. When compared to unprotected CS samples, the quality of cfRNA improved in 91.0% (101/111) of cases, and the failure rate (indicated by CT values greater than 27.0 or undetected amplification curves in RT‐qPCR) significantly decreased (4.3% vs. 38.0%) in PS:CS = 1:1 group. The preservation protocol recommended in this study offers a novel strategy for simultaneously achieving higher accuracy in detecting SNV/InDels and fusions in CS samples through cfDNA and cfRNA extraction and double testing. To the best of our knowledge, this is the first report to test cfDNA and protected cfRNA originated from CS samples simultaneously for the detection of driver genes mutations in NSCLC.

In the present study, a total of 84 patients with NSCLC were enrolled, and the cfDNA and cfRNA extracted from CS samples were analyzed for 11 driver genes changes in NSCLC. The results revealed that for RT‐qPCR for multi‐gene mutations detection, the overall sensitivity and specificity of patients with malignant CS samples were 93.8% and 100%. In RNA‐based tests, the sensitivity of malignant CS samples was 100%. And among all the 10 patients with targeted therapy, the ORR was 60% and the DCR was 100%. These results suggest that the stepwise protocol recommended in this study makes malignant CS samples applicable for detecting 11 driver genes in NSCLC using cfDNA & cfRNA simultaneously. Especially for cfRNA based detection, including ALK, ROS1, RET and MET ex14 skipping, the protocol ensures a higher sensitivity and accuracy than that of previous published literatures.^7^, ^29^, ^30^

Notably, CS preparation from diverse cytological samples plays a pivotal role for successful molecular detection in this study. We divide CS samples into two categories based on their mucus content with different preparation procedures. And for hemorrhagic cytological samples, lysate solution of red blood cells should be added into the sediment after CS sample collection in case decomposition of cell‐free nucleic acid. This protocol is both economical and practical, with straightforward instructions for CS preparation.

In addition, the quality control (QC) of protected cfRNA is crucial for the RNA‐based driver genes detection. An evaluation system of CT values for cfRNA's QC was established with four tiers (CT value ≤23.0, 25.0, 27.0 and >27.0 or no amplification curves detection). When CT value ≤25.0, the result of fusion genes detection are reliable. CT value between 25.0 and 27.0, the testing result has the possibility of false‐negative.

This study has some limitations. First, due to the extremely rare mutation ratio, NTRK1/2/3 fusions were not found.^31^, ^32^ Also, we are not able to make sure the certain variant type of fusion and specific mutations site, because the PCR reagents applied in this study, each PCR reaction hole contains more than one variant type. It is the technical limitation. Second, the results of CS from needle aspirations were problematic, with 50% (4/8) failure cases, this suggests that the preparing of CS for needle aspiration need to be refined further. Other and our published studies have indicated that using physiological saline or RPMI 1640 for CS preparation can suffice for genotyping at cfDNA level.^8^, ^22^ It is likely that the high failure rate could be influenced by the chemical property of fixation solution used in this study and resulted in the lower release of cfDNA and cfRNA from needle biopsy tissue. Therefore, we will choose physiological saline to prepare for the supernatant of core needle aspiration samples in further studies. Meanwhile, 15% (3/20) BALF samples were failed. The failure cases may influenced by the low recovery rate of BALF, <5 mL BALF was collected in three failure cases. The amount of BALF collection ≥5 mL is recommended for cfDNA and cfRNA double detection. Third, demographic distributions of age, gender, and stage could introduce bias in this study with a high proportion of age ≥65 (69.0%), male (60.7%) and Stage IIIA–IV (79.8%). In addition, the Pan Lung Cancer PCR Panel kit applied in this study contains the commonest variants, including 231 mutations (112 in DNA level, 119 in RNA level). Rare or novel mutations may not include in this kit. The occurrence of the false negative could not be ruled out.

In addition, comparative experiments between RT‐qPCR and digital droplet PCR (ddPCR) (TargetingOne Corporation) were conducted among 84 cytological samples for multiple driver gene mutations detection. There was a high degree of agreement in cfDNA level, while in cfRNA level, the results of ddPCR showed a relatively low sensitivity (90.9%) and specificity (87.9%), and the overall concordance rate was 88.6%. Based on the above data, the technique of ddPCR in this study was immature and we will launch for the technical improvement for ddPCR in the future, especially for increasing the specificity of cfRNA‐based multiple gene mutations detection.

Overall, this study validated the feasibility of cfDNA and cfRNA extraction and double detection for driver genes mutations in malignant CS samples in NCSLC patients. We provide the PS for cfRNA protection and broaden the CS samples categories for cfRNA‐based testing, including body cavity effusion, sputum, BALF and needle aspiration. In addition, pathological evaluation shows that morphology of cells is well maintained. The recommended protocol enhances a balance of cytopathological diagnosis and driver genes mutations detection. Meanwhile, the accuracy of cfRNA testing has been improvement significantly.

## AUTHOR CONTRIBUTIONS

**Yidan Ma:** Data curation (lead); formal analysis (lead); investigation (lead); visualization (lead); writing – original draft (lead). **Yifei Wang:** Data curation (supporting); investigation (equal). **Lei He:** Investigation (equal). **Jun Du:** Investigation (equal). **Lin Li:** Resources (equal). **Zhixin Bie:** Resources (equal). **Yuanming Li:** Resources (equal). **Xiaomao Xu:** Resources (equal). **Wei Zhou:** Resources (equal). **Xiaonan Wu:** Resources (equal). **Li Yang:** Investigation (supporting). **Jing Di:** Investigation (supporting). **Chenyang Li:** Investigation (supporting). **Xiaoguang Li:** Resources (equal); supervision (equal). **Dongge Liu:** Conceptualization (equal); resources (equal); supervision (equal). **Zheng Wang:** Conceptualization (lead); funding acquisition (lead); supervision (lead); writing – review and editing (lead).

## FUNDING INFORMATION

The National High Level Hospital Clinical Research Funding (BJ‐2219‐195), Grant/Award Number: BJ‐2219‐195. the National High Level Hospital Clinical Research Funding (BJ‐2023‐090), Grant/Award Number: BJ‐2023‐090. Ethical approval statement. This study was approved by the Beijing Hospital Institution Review Board (Approval number:2020BJYYEC‐065–02). All participants provided written informed consent.

## Supporting information


Figure S1.

Figure S2.

Table S1.

Data S1.

## Data Availability

Some or all data generated or used during the study are available from the corresponding authorby request.
