# Supplementary material for: Preservation of cfRNA in cytological supernatants for cfDNA & cfRNA double detection in non‐small cell lung cancer patients
Source: Cancer Med. 2024 Sep 5;13(17):e70197. doi: 10.1002/cam4.70197 (PMC11375324; doi:10.1002/cam4.70197)

Figure S1 Amplification curves of protected cfRNA in the groups with various proportions of PS:CS by RT-qPCR.

The results of CT value were 23.58 in PS:CS=0:1, 20.65 in PS:CS=1:1, 21.91 in PS:CS=1:2, 22.90 in PS:CS=1:3

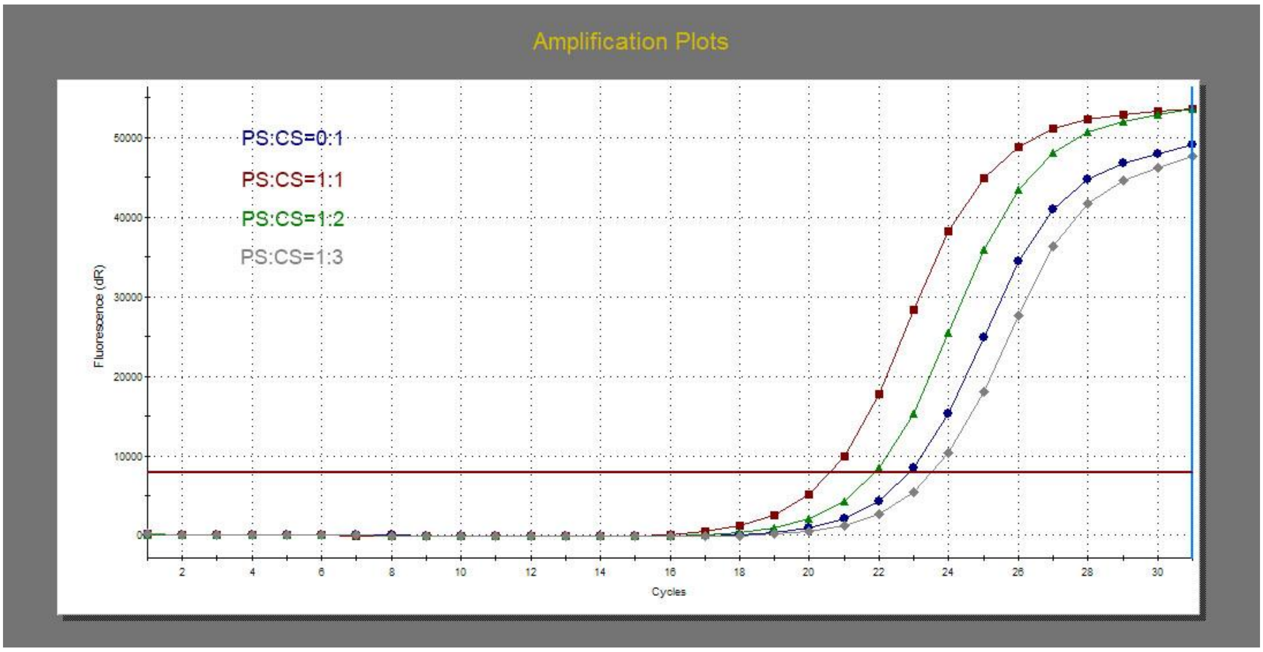

Supplement: Supplementary file 1 — Figure S1. [file CAM4-13-e70197-s002.pdf]
