# Supplementary material for: Preservation of cfRNA in cytological supernatants for cfDNA & cfRNA double detection in non‐small cell lung cancer patients
Source: Cancer Med. 2024 Sep 5;13(17):e70197. doi: 10.1002/cam4.70197 (PMC11375324; doi:10.1002/cam4.70197)

Figure S2 One case with ROS1 gene fusion positive was detected from sputum CS sample
HEX curve was the internal control. FAM curve was the ROS1 gene

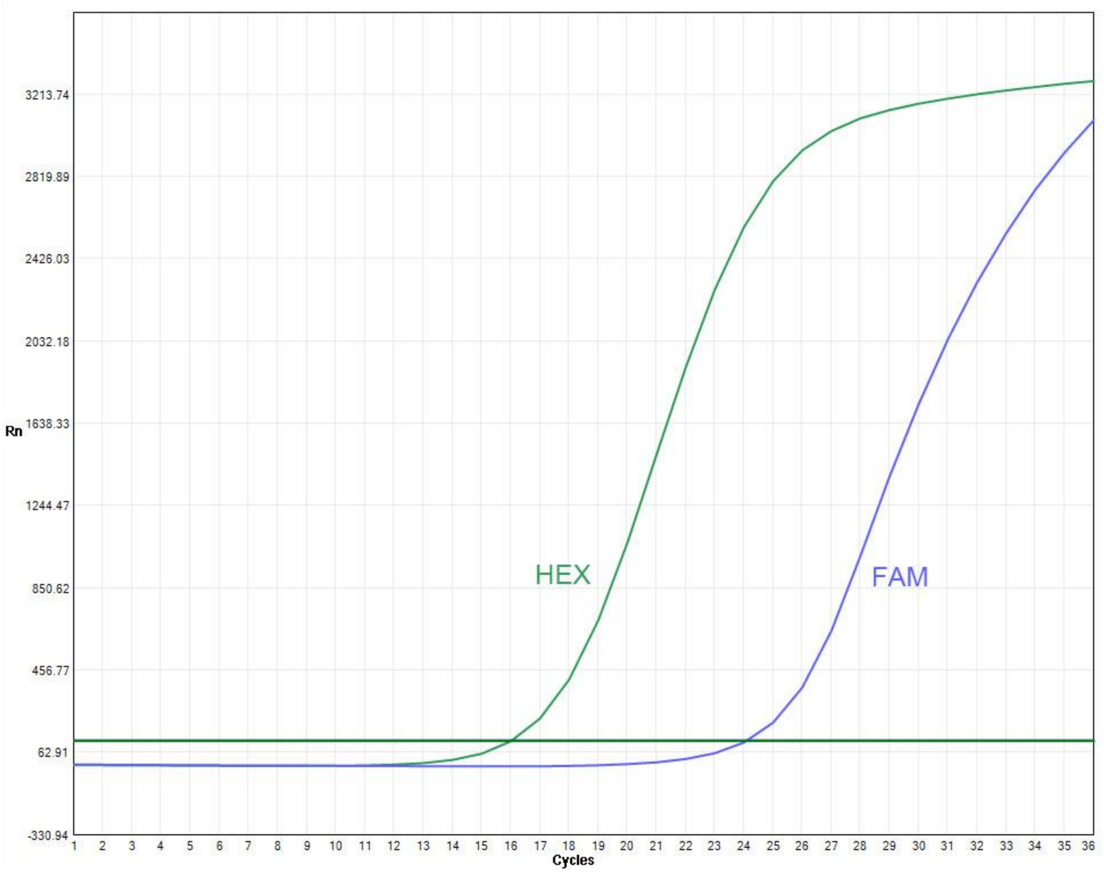

Supplement: Supplementary file 2 — Figure S2. [file CAM4-13-e70197-s001.pdf]
