# Supplementary material for: Preservation of cfRNA in cytological supernatants for cfDNA & cfRNA double detection in non‐small cell lung cancer patients
Source: Cancer Med. 2024 Sep 5;13(17):e70197. doi: 10.1002/cam4.70197 (PMC11375324; doi:10.1002/cam4.70197)
Supplement: Supplementary file 3 — Table S1. [file CAM4-13-e70197-s004.docx]

Table S1 Clinicopathologic characteristics of of 7 samples with failed detection

| #Case | Age | Sex | Stage | Sample | Patients’ treatment status |
| --- | --- | --- | --- | --- | --- |
| #1 | 79 | male | IV | BALF | Treatment naive |
| #2 | 77 | female | IA | BALF | Treatment naive |
| #3 | 76 | male | IV | BALF | Treatment naive |
| #4 | 55 | male | IV | Needle aspiration | Treatment naive |
| #5 | 83 | male | IV | Needle aspiration | Treatment naive |
| #6 | 70 | female | IIIA | Needle aspiration | Treatment naive |
| #7 | 88 | female | IA | Needle aspiration | Treatment naive |

Table S2 Clinicopathologic characteristics of samples with genes alternations in cfRNA level

| #Case | Age | Sex | Stage | Genes alternations | Sample |
| --- | --- | --- | --- | --- | --- |
| #1 | 92 | male | IV | MET ex14 skipping | Body cavity effusion |
| #2 | 46 | male | IV | EML4-ALK | Body cavity effusion |
| #3 | 71 | male | IV | RET | Body cavity effusion |
| #4 | 72 | male | IV | ROS1 | Sputum |
| #5 | 39 | male | IV | EML4-ALK | Body cavity effusion |
| #6 | 43 | male | IV | EML4-ALK | Body cavity effusion |
| #7 | 61 | male | IV | EML4-ALK | Body cavity effusion |
| #8 | 59 | male | IV | EML4-ALK | Body cavity effusion |
| #9 | 64 | female | IV | EML4-ALK | Body cavity effusion |
| #10 | 49 | female | / | EML4-ALK | Needle aspiration |
| #11 | 52 | female | / | MET ex14 skipping | Needle aspiration |
