# Supplementary material for: Preservation of cfRNA in cytological supernatants for cfDNA & cfRNA double detection in non‐small cell lung cancer patients
Source: Cancer Med. 2024 Sep 5;13(17):e70197. doi: 10.1002/cam4.70197 (PMC11375324; doi:10.1002/cam4.70197)
Supplement: Supplementary file 4 — Data S1. [file CAM4-13-e70197-s003.docx]

**Supplementary material for materials and methods**

Cytological samples were collected into 50 ml centrifuge tubes and stored at 4℃ until samples were processed within 4 hours. Based on their mucus content, they were categorized into two groups (Figure 1B). For sputum and BALF containing mucus, an initial mucolysis treatment was applied and adding fixation liquid to 3-5 ml samples. The fixation liquid was composed of 2% polyethylene glycol 400 (Aladdin) in 50% Ethyl Alcohol (fixation solution), combined with 0.5mol/L dithiothreitol (Sigma) in a 9:1 ratio (laboratory developed, patent application number 202310823963.0, China National Intellectual Property Administration). After vortex 5 minutes, the mucus content within samples could be lysed thoroughly. For samples without mucus, 20 ml needle aspiration samples suspended in the fixation liquid and 20-50 ml body cavity effusions were collected in 50 ml centrifuge tube. Subsequently, all cytological samples were centrifuged at 2000 rpm for 5 minutes. After centrifuging, the CS was collected and divided into different PS:CS proportions respectively (Figure 1A). The processed samples were stored at -80℃ for cfDNA and cfRNA analysis within 30 days. The sediments of cytological samples were re-suspended in PreservCyt Solution (Hologic Inc.) to prepare liquid-based slides for pathological evaluation and when malignant cells were found, the CS samples were taken or further cfDNA & cfRNA evaluation and genotyping.
